# A comparative plastome approach enhances the assessment of genetic variation in the *Melilotus* genus

**DOI:** 10.1186/s12864-024-10476-y

**Published:** 2024-06-03

**Authors:** Pan Xu, Minghui Meng, Fan Wu, Jiyu Zhang

**Affiliations:** State Key Laboratory of Grassland Agro-ecosystems, Key Laboratory of Grassland Livestock Industry Innovation, Ministry of Agriculture and Rural Affairs, Engineering Research Center of Grassland Industry, College of Pastoral Agriculture Science and Technology, Ministry of Education, Lanzhou University, Lanzhou, 730000 China

**Keywords:** *Melilotus*, Chloroplast genome, Evolution, Inversion

## Abstract

**Background:**

*Melilotus*, a member of the Fabaceae family, is a pivotal forage crop that is extensively cultivated in livestock regions globally due to its notable productivity and ability to withstand abiotic stress. However, the genetic attributes of the chloroplast genome and the evolutionary connections among different *Melilotus* species remain unresolved.

**Results:**

In this study, we compiled the chloroplast genomes of 18 *Melilotus* species and performed a comprehensive comparative analysis. Through the examination of protein-coding genes, we successfully established a robust phylogenetic tree for these species. This conclusion is further supported by the phylogeny derived from single-nucleotide polymorphisms (SNPs) across the entire chloroplast genome. Notably, our findings revealed that *M. infestus*, *M. siculus*, *M. sulcatus*, and *M. speciosus* formed a distinct subgroup within the phylogenetic tree. Additionally, the chloroplast genomes of these four species exhibit two shared inversions. Moreover, inverted repeats were observed to have reemerged in six species within the IRLC. The distribution patterns of single-nucleotide polymorphisms (SNPs) and insertions/deletions (InDels) within protein-coding genes indicated that *ycf1* and *ycf2* accumulated nonconservative alterations during evolutionary development. Furthermore, an examination of the evolutionary rate of protein-coding genes revealed that *rps18*, *rps7*, and *rpl16* underwent positive selection specifically in *Melilotus*.

**Conclusions:**

We present a comparative analysis of the complete chloroplast genomes of *Melilotus* species. This study represents the most thorough and detailed exploration of the evolution and variability within the genus *Melilotus* to date. Our study provides valuable chloroplast genomic information for improving phylogenetic reconstructions and making biogeographic inferences about *Melilotus* and other Papilionoideae species.

**Supplementary Information:**

The online version contains supplementary material available at 10.1186/s12864-024-10476-y.

## Background

With the extensive evolution of plants, each plant has a distinct origin and diverse evolutionary history that has changed its genetic makeup compared to that of related species, leading to various physiological and phenotypic differences [1, 2]. With advancements in technology, plant genome sequencing has become more affordable and accessible. By analysing sequences and using comparative genomics methods [3], researchers can assess biological events such as positive selection, genetic diversity, chromosome structure variation and polyploidy, which have become hotspots of biological research in recent years [4, 5]. The maternal inheritance of chloroplast genomes, characterized by their unique attributes [6], offers a convenient and dependable avenue for elucidating plant evolution and genetic interconnections among closely related species [7]. In contrast to intricate and extensive nuclear genomes, chloroplast genomes, which are typically smaller than 200 kb, exhibit moderate nucleotide substitution rates [8], rendering them amenable to sequencing using both next-generation sequencing and single-molecule long-read sequencing technologies [9]. The analysis of complete chloroplast genome sequences offers a substantial amount of valuable information, including insights into structural variations, gene losses, and single-base mutations. This information can be utilized to enhance our understanding of evolutionary distinctions, investigate genetic diversity, and construct detailed phylogenetic trees [10, 11]. Comparative chloroplast genome examination has proven to be an effective method for identifying evolutionary relationships at the species level [12–14].

Notably, the family Fabaceae is the third largest family among angiosperms [15]. Species of this family are grown for food and feed, as well as are ideal models for studying classification, diversity and genetic evolution. Based on the analysis of *matK* sequences and a comprehensive sampling approach, it is evident that the legume family can be distinctly categorized into six monophyletic subfamilies [16]. Notably, Papilionoideae, which comprises a vast assemblage of more than 14,000 species, stands out as the subfamily with the highest species diversity. Furthermore, within Papilionoideae, a more intricate classification reveals six distinct clades, with the largest one being IRLC (inverted repeat lacking clade) [17]. This particular clade encompasses approximately 4000 species spanning 52 genera, including *Melilotus*. At present, the genomes of multiple species of Fabaceae, such as soybean [18], alfalfa [19], pea [20], and red clover [21], have been sequenced, providing a basis for comparative genome studies and evolutionary history examination [22]. However, while *Melilotus* is an important Papilionoideae genus with 19 species [23], information about the genome of the *Melilotus* spp (also called sweet clover) remains scarce [24–26]; however, *Melilotus* members are widely planted in global livestock areas due to their high yield and resistance to abiotic stress [27, 28].

*Melilotus*, a significant member of the Leguminosae family, consists of 19 annual or biennial species. *Melilotus* spp serves as a rotational crop primarily utilized for forage production, soil enhancement, and a nectar source. *Melilotus* spp. are renowned for their remarkable resilience to drought, cold, and high-salinity conditions [29, 30]. Despite the application of various molecular marker-based techniques to evaluate genetic diversity in *Melilotus* species, the evolutionary connections among these species remain ambiguous [31–34]. Here, we report the assemblies and gene annotations of the chloroplast genomes of 18 *Melilotus* species and compare their genome sequences to identify structural variants. Phylogenetic trees are constructed to illustrate the evolutionary relationships of *Melilotus* by based on single-copy genes and single- nucleotide polymorphisms (SNPs). The chloroplast genomes of the 18 Melilotus species assembled in this study will be a useful resource for genetic studies and taxonomy.

## Results

### Sequencing of plant samples

We downloaded the whole-genome sequencing data of 18 species of sweet clover from the National Center for Biotechnology Information Short Read Archive, including *M. albus*, *M. officinalis*, *M. altissimus*, *M. dentatus*, *M. elegans*, *M. hirsutus*, *M. indicus*, *M. infestus*, *M. italicus*, *M. polonicus*, *M. segetalis*, *M. siculus*, *M. speciosus*, *M. spicatus*, *M. suaveolens*, *M. sulcatus*, *M. tauricu* and *M. wolgicus*. The short reads from whole-genome sequencing of all species were generated using the Illumina HiSeq 2500 platform. The total number of clean bases ranged from 5.0 Gb to 9.7 Gb, with GC contents ranging from 34.75 to 37.14%. This sequencing depth represented approximately 10-fold coverage of the entire genome. To verify the reliability of the assembly, 17 out of the 18 species were selected and newly sequenced, with the total number of clean bases ranging from 14.0 Gb to 58.0 Gb (Additional file 2: Table S1). Subsequently, the chloroplast genomes were assembled again based on the new data and compared with the previous assembly for each species.

### Chloroplast genome assembly and annotation

**Fig. 1:**
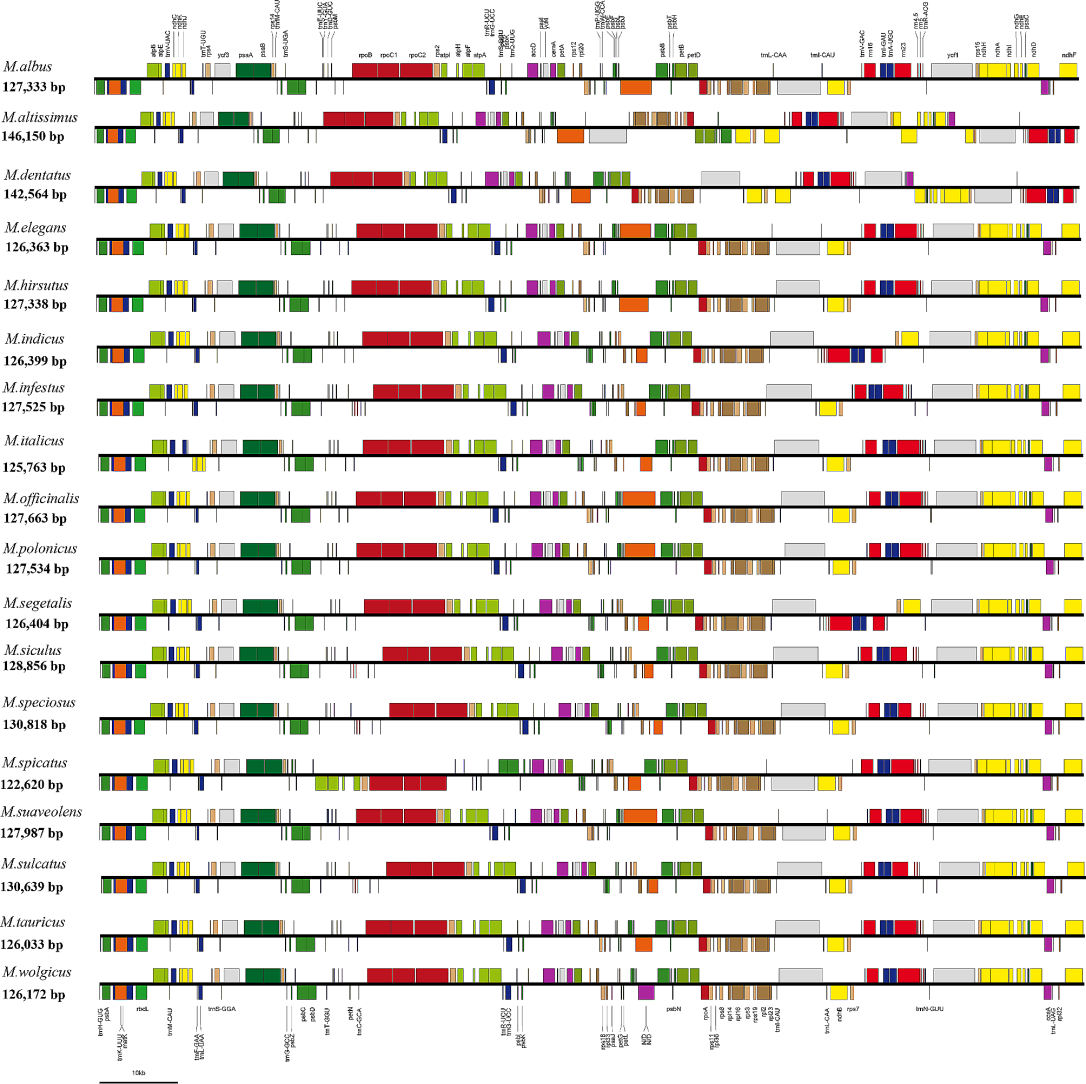
Gene features of 18 assembled chloroplast genomes. The genes drawn above the line are on the positive strand, and the genes drawn under the line are located on the negative strand

The lengths of the chloroplast genomes of the assembled species ranged from 122,620 to 146,150 base pairs (bp). Additionally, the GC content of these genomes varied between 33.56% and 34.53%. Notably, no gaps were detected in any of the genomes. Each of the 18 species underwent comprehensive annotation, resulting in the identification of approximately 110 functional genes. These genes included 76–79 protein-coding genes (PCGs), 30–35 transfer RNA (tRNA) genes, and 4–8 ribosomal RNA (rRNA) genes (as presented in Table 1). Furthermore, the majority of these species possessed a single copy of the inverted repeat (IR). The arrangement of genes on the chromosomes exhibited remarkable consistency across all chloroplast genomes, with only a few potential inversions observed (as depicted in Fig. 1). Inverted repeat regions were detected only in *M. altissimus*, *M. dentatus*, *M. speciosus*, *M. infestus*, *M. siculus*, and *M. sulcatus*, and the length of the IR region varied greatly from 3,518 bp ~ 18,439 bp (Table 1). Due to duplication and the emergence of IR regions, 5 genes were found to have two copies, including one protein-coding gene (*psbM*), two tRNA genes (*trnN-GUU* and *trnR-ACG*), two rRNA genes (*rrn5* and *rrn4.5*) in *M. infestus*, *M. siculus*, *M. sulcatus*, and *M. speciosus*; 10 genes were found to have two copies in *M. dentatus*, including one protein-coding gene (*ycf1*), five tRNA genes (*trnA-UGC*, *trnI-GAU*, *trnN-GUU*, *trnR-ACG*, and *trnV-GAC*), and four rRNA genes (*rrn16*, *rrn23*, *rrn4.5*, and *rrn5*); and 13 genes were found to have two copies in *M. altissimus*, including four protein-coding genes (*ycf1, rps15, ndhH*, and *ndhA*), five tRNA genes (*trnA-UGC*, *trnI-GAU*, *trnN-GUU*, *trnR-ACG*, and *trnV-GAC*), and four rRNA genes (*rrn16*, *rrn23*, *rrn4.5*, and *rrn5*).

**Table 1:** Features summary of of the 18 *Melilotus* species chloroplast genomes

Species	Length	IR length	GC%	Number of genes			
				PCGs	tRNA	rRNA	Total
*M. albus*	127,333	-	33.65	76	30	4	110
*M. altissimus*	146,150	18,439	34.53	79	35	8	122
*M. dentatus*	142,564	15,494	34.50	77	35	8	120
*M. elegans*	126,363	-	33.67	76	30	4	110
*M. hirsutus*	127,338	-	33.57	76	30	4	110
*M. indicus*	126,399	-	33.86	76	30	4	110
*M. infestus*	127,525	3,528	34.23	77	32	6	115
*M. italicus*	125,763	-	33.99	76	30	4	110
*M. officinalis*	127,663	-	33.63	76	30	4	110
*M. polonicus*	127,534	-	33.83	76	31	4	111
*M. segetalis*	126,404	-	33.86	76	30	4	110
*M. siculus*	128,856	3,670	34.33	77	32	6	115
*M. speciosus*	130,818	4,317	34.10	76	32	6	114
*M. spicatus*	122,620	-	33.94	76	30	4	110
*M. suaveolens*	127,987	-	33.56	76	30	4	110
*M. sulcatus*	130,639	3,876	34.09	77	32	6	115
*M. tauricus*	126,033	-	33.79	76	30	4	110
*M. wolgicus*	126,172	-	33.80	76	30	4	110

### Comparison of chloroplast genomes within *Melilotus*

**Fig. 2:**
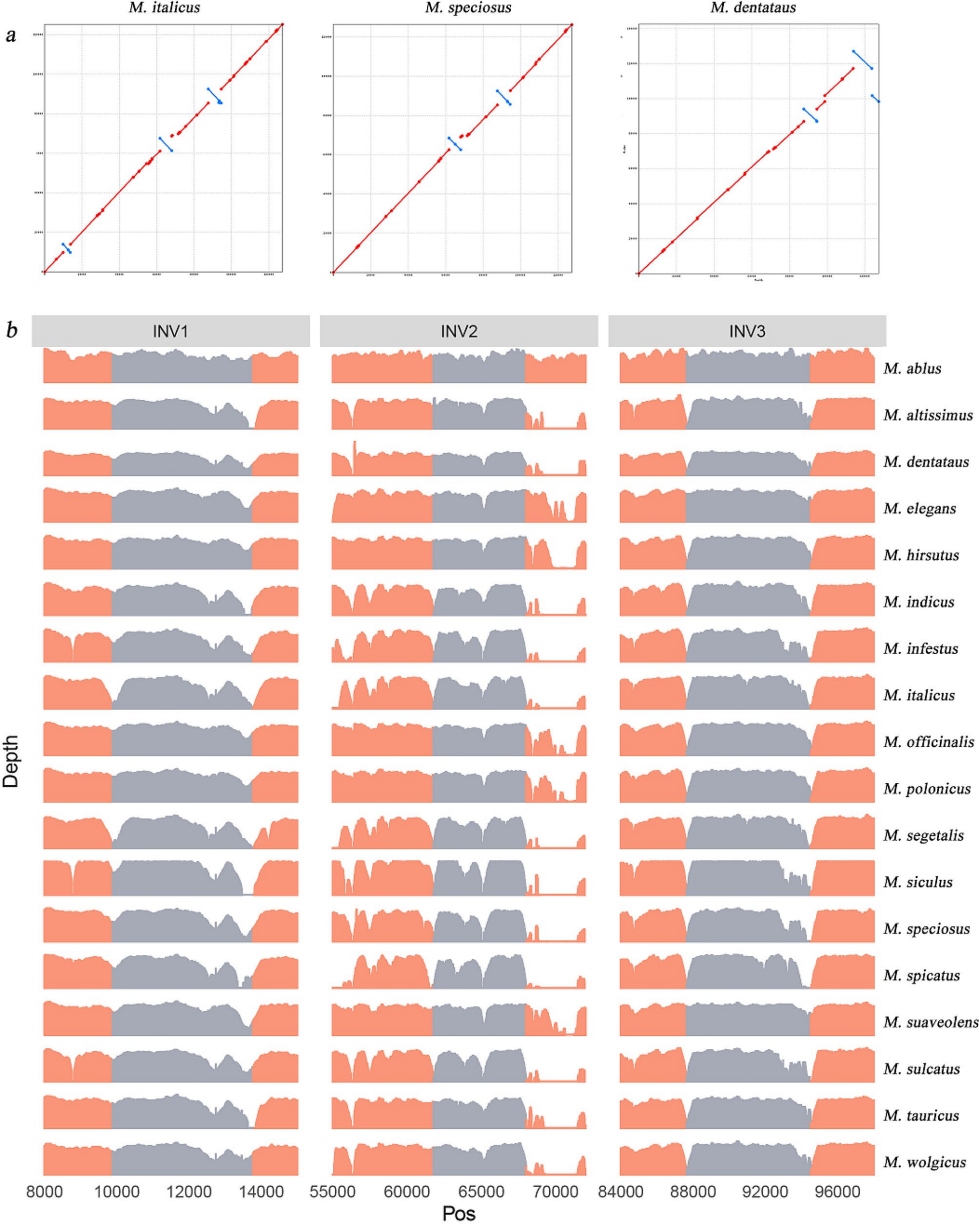
Structural variations between *M. albus* and other species. Collinear plot between *M. italicus*, *M. speciosus*, *M. dentatus* and *M. albus* (**a**). Depth of Illumina short reads mapped to *M. albus* near three INVs (**b**)

According to the collinearity between *M. albus* and other *Melilotus* chloroplast genomes (Additional file 1: Fig. S1), three main inversions (INV1, INV2 and INV3) were detected at locations near 9,890 to 13,774 bp, 61,784 to 67,994 bp and 87,643 to 94,511 bp, with fragment lengths of 3,884 bp, 6,210 bp and 6,868 bp, respectively (Fig. 2a). The decline in mapping depths near the start and end of potential inversion regions during the mapping of short reads from whole-genome resequencing data to the *M. albus* genome may support the presence of an inversion (Fig. 2b). We found that the *ndhC*, *ndhK* and *ndhJ* genes were contained in INV1; the *rps12*, *rpl20*, *rps18*, *rpl33*, *psaJ*, *petG*, *petL*, *psbE*, *psbF*, *psbL* and *psbJ* genes were contained in INV2; and only the *ycf2* gene was contained in INV3. The species *M. italicus* had all three INVs; *M. infestus*, *M. speciosus*, *M. siculus*, and *M. sulcatus* had both INV2 and INV3; and no INVs were detected in *M. elegans* or *M. suaveolens* (Table 2).

**Table 2:** Three main inversions among the species of sweet clovers

ID	Start	End	Species	Inversion PCGs
INV1	9,890	13,774	*M. italicus*	*ndhC*, *ndhK*, *ndhJ*
INV2	61,784	67,994	*M. indicus*, *M. infestus*, *M. italicus*, *M. segetalis*, *M. siculus*, *M. speciosus*, *M. spicatus*, *M. sulcatus*	*rps12*, *rpl20*, *rps18*, *rpl33*, *psaJ*, *petG*, *petL*, *psbE*, *psbF*, *psbL*, *psbJ*
INV3	87,643	94,511	*M. dentatus*, *M. hirsutus*, *M. indicus*, *M. infestus*, *M. italicus*, *M. polonicus, M. officinalis*, *M. segetalis*, *M. siculus*, *M. speciosus*, *M. sulcatus*, *M. tauricus*, *M. wolgicus*	*ycf2*

### Phylogenetic analysis

**Fig. 3:**
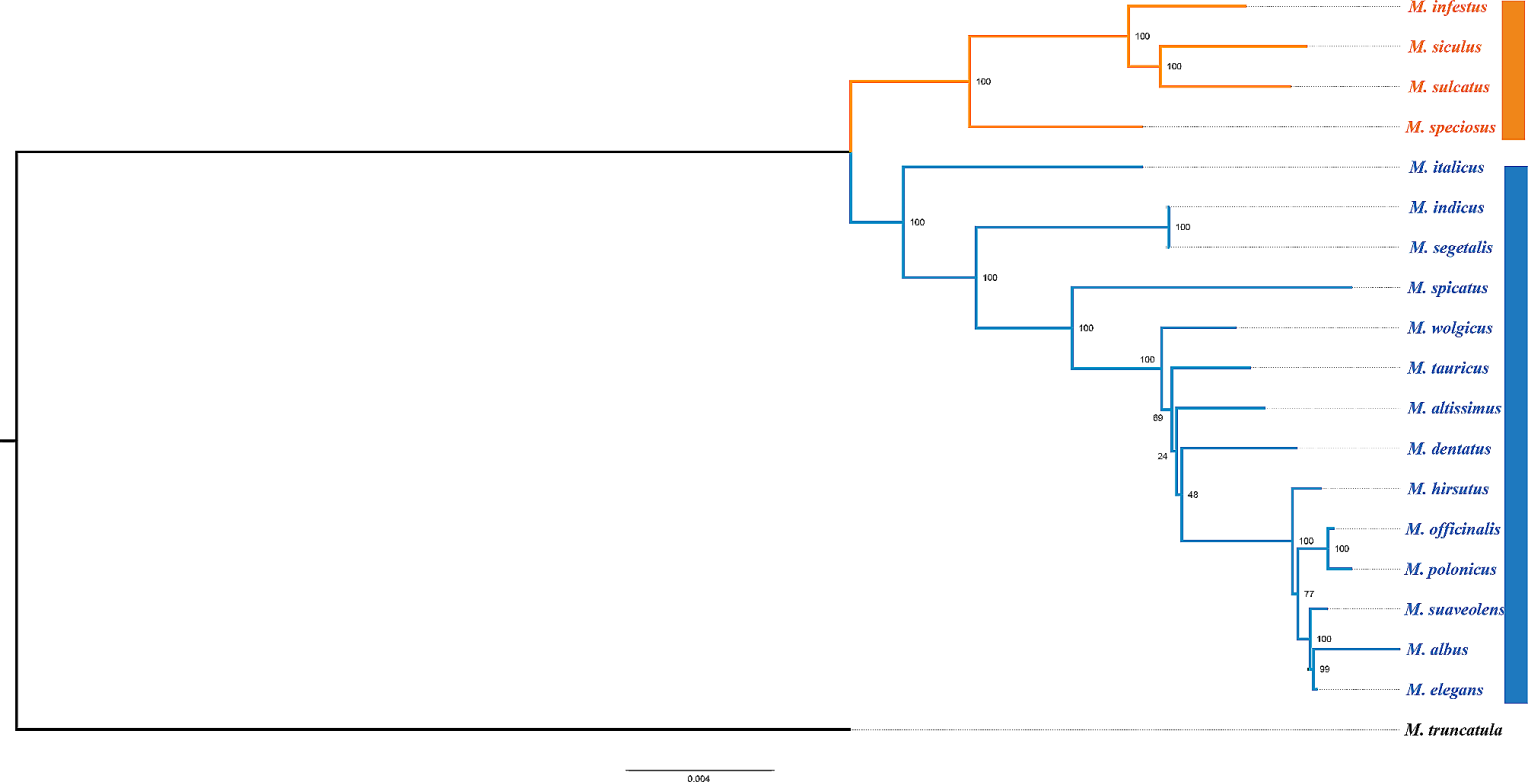
Phylogenetic tree was constructed using single copy genes of 18 species by IQ-Tree, the size of green circle on the branch represented the value of bootstrap

We constructed a phylogenetic tree of 18 *Melilotus* species utilizing 76 protein-coding genes through IQ-TREE and MrBayes. *Medicago truncatula* was employed as an outgroup in the analysis (Fig. 3 and Additional file 1: Fig. S2). Additionally, we constructed a phylogenetic tree based on the complete chloroplast genome sequences (Additional file 1: Fig. S3). The topologies of the three phylogenetic trees were consistent, revealing that all 18 species could be categorized into two distinct subgroups. One subgroup consisted of *M. infestus*, *M. siculus*, *M. sulcatus* and *M. speciosus*. All species within this subgroup exhibited INV2 and INV3, in comparison to the *M. albus* reference. Of particular significance, they all possessed inverted repeat regions. All remaining species, including two important ones, *M. albus* and *M. officinalis*, constitute the other subgroup. The interspecific relationships revealed that *M. officinalis* diverged earlier than *M. albus*. Among the other species, *M. indicus* and *M. segetalis* shared a common ancestor and exhibited the most robust collinearity among the 18 chloroplast genomes (Fig. 1).

### Nucleotide substitution rates

**Fig. 4:**
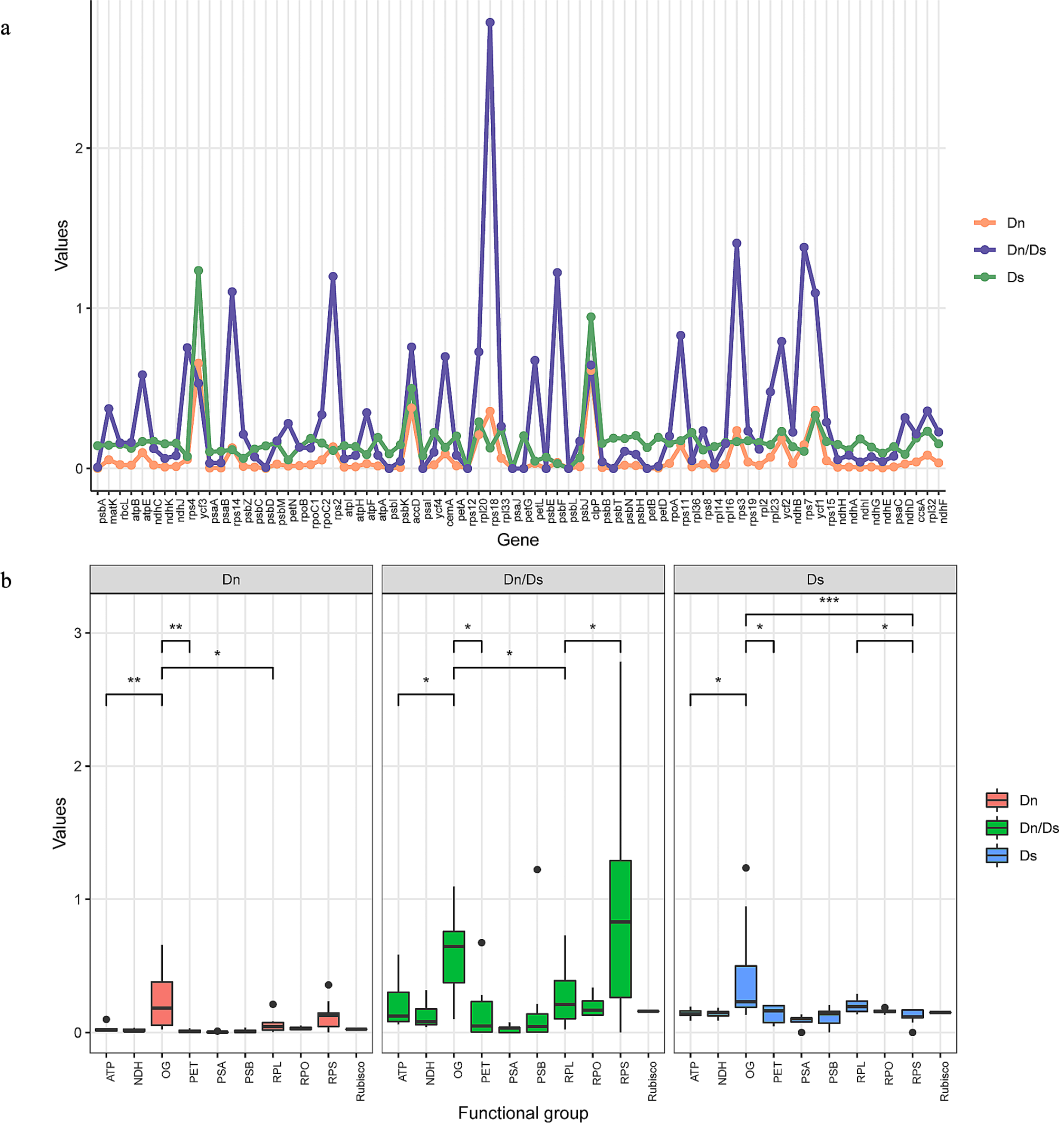
Dn (Nonsynonymous), Ds (synonymous) and Dn/Ds of each PCG (**a**). Dn, Ds and Dn/Ds of nine functional groups (**b**)

Substitution rates, such as synonymous substitution (Ds), nonsynonymous substitution (Dn) and Dn/Ds, were estimated for the 76 PCGs to detect evolutionary rate heterogeneity and to represent different selection regimes acting on PCGs (Table 3). Among the 76 PCGs, *accD*, *clpP*, and *ycf3* had relatively high Ds and Dn values simultaneously, while *rps18*, *rps7*, and *rpl16* exhibited high Dn/Ds values (> 1), indicating that they have experienced positive selection. These three genes were located within the inversion regions, suggesting that these specific regions may have played a role in the elevated Dn/Ds values in *Melilotus* (Fig. 4a). These genes were further categorized into ten groups, comprising nine functional groups and one group of other genes (OG, Table 3). Evolutionary rates were then compared across these groups. Both the OG and RPS categories(ribosomal protein small subunit) exhibited the highest median values for Dn and Dn/Ds, with a moderately high median Ds value. Notably, the Dn, Dn/Ds, and Ds values in the OG category were significantly greater than those in the ATP and NDH category. Genes encoding subunits involved in photosynthetic processes, such as Photosystems I and II (PSA and PSB), ATP synthase (ATP) and the cytochrome b6f complex (PET), exhibited lower rates of nucleotide substitution than genes in other functional groups. The Rubisco large subunit (Rubisco) genes displayed moderately lower Dn, Ds and Dn/Ds values than did the other groups (Fig. 4b).

**Table 3:** Plastid genes and their functional groups included in analyses

Functional groups	Genes
Photosystem I (PSA)	*psaA*, *psaB*, *psaC*, *psaI*, *psaJ*
Photosystem II (PSB)	*psbA*, *psbB*, *psbC*, *psbD*, *psbE*, *psbF*, *psbH*, *psbI*, *psbJ*, *psbL*, *psbM*, *psbN*, *psbT*, *psbZ*
Cytochrome B6f complex (PET)	*petA*, *petB*, *petD*, *petG*, *petF*, *petN*
ATP synthase (ATP)	*atpA*, *atpB*, *atpE*, *atpF*, *atpH*, *atpI*
Rubisco large subunit (Rubisco)	*rbcL*
RNA polymerase (RPO)	*rpoA*, *rpoB*, *rpoC1*, *rpoC2*
Ribosomal proteins large subunit (RPL)	*rpl2*, *rpl14*, *rpl16*, *rpl20*, *rpl22*, *rpl23*, *rpl32*, *rpl33*, *rpl36*
Ribosomal proteins small subunit (RPS)	*rps2*, *rps3*, *rps4*, *rps7*, *rps8*, *rps11*, *rps12*, *rps14*, *rps15*, *rps18*, *rps19*
NADH dehydrogenase (NDH)	*ndhA*, *ndhB*, *ndhC*, *ndhD*, *ndhE*, *ndhF*, *ndhG ndhH ndhI ndhJ ndhK*
Other genes (OG)	
Conserved coding frame	*ycf1*, *ycf2*, *ycf3*, *ycf4*
Acetyl-CoA-carboxylase	*accD*
ATP-dependent protease	*clpP*
Cytochrome c biogenesis	*ccsA*
Membrane protein	*cemA*
Maturase	*matK*

### Variant calling and construction of the SNP phylogenetic tree

**Fig. 5:**
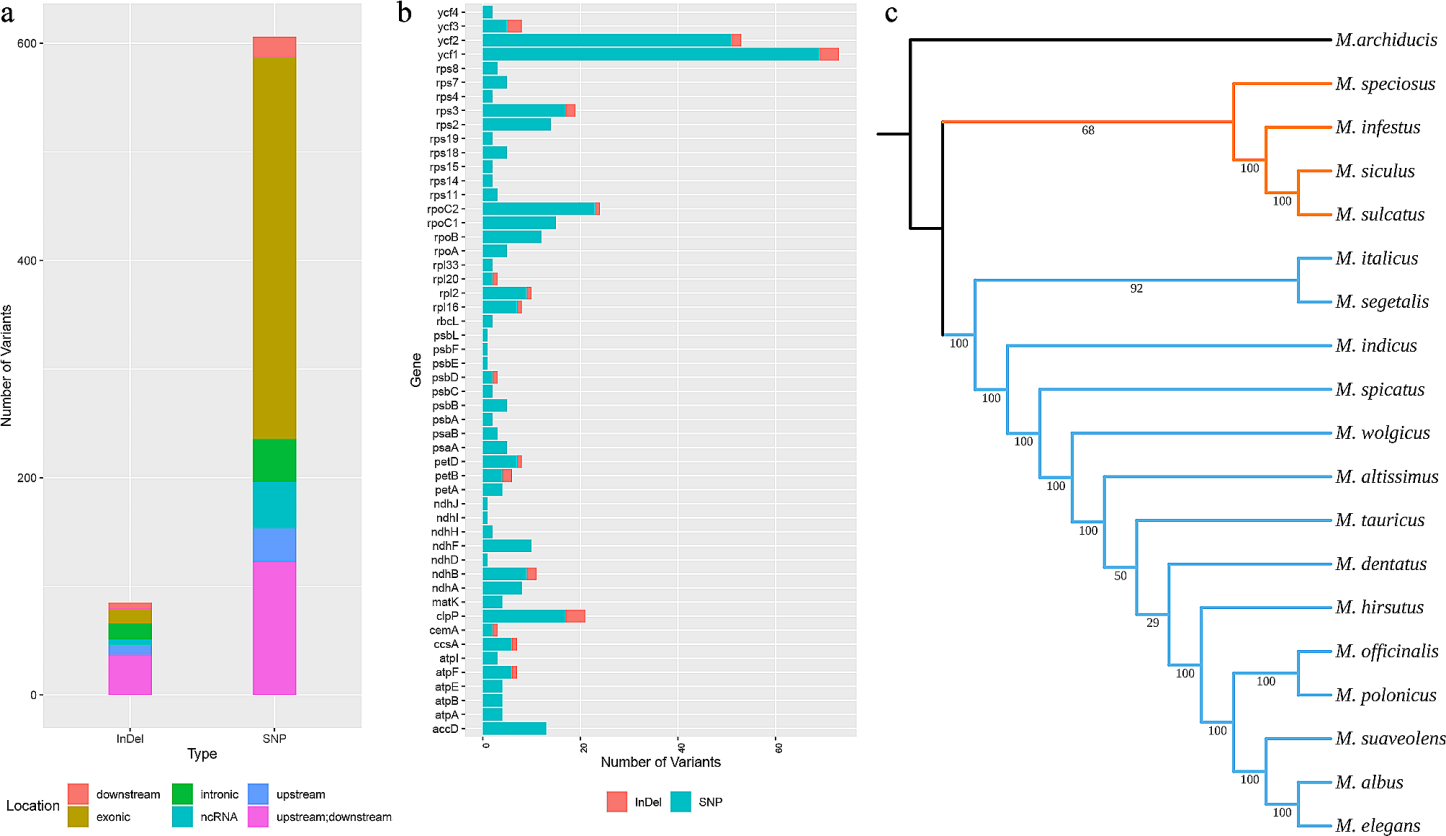
The distributions of mutations located in the chloroplast genome (**a**). Numbers of SNPs and InDels in each PCG (**b**). Phylogenetic tree was constructed using SNPs (**c**)

Variants were identified using the reference genome of *M. albus*, ultimately revealing a total of 606 SNPs and 85 InDels. There were 364 mutations located in coding sequences (351 SNPs and 13 InDels), including 117 synonymous and 233 nonsynonymous mutations. Among the other mutations, 172 SNPs and 52 InDels were located upstream and downstream of genes, and 83 SNPs and 20 InDels were located in noncoding RNA or introns (Fig. 5a and Additional file 2: Table S2). The distribution of SNPs and InDels on PCGs suggested that genes such as *ycf1* and *ycf2* have undergone nonconservative evolution. (Fig. 5b and Additional file 3: Table S3). Only the SNPs from these mutations were used to construct the phylogenetic tree, which had a similar structure to the tree constructed using single-copy genes. *M. speciosus, M. infestus, M. siculus*, and *M. sulcatus* formed a distinct group with the greatest genetic distance from *M. albus*. However, some differences were detected between the relationships of *M. atissimus*, *M. dentatus* and *M. tauricus* in the two trees (Fig. 5c).

## Discussion

The results presented in this study mark the first instance of utilization of the entire chloroplast genome to investigate genetic variation across 18 *Melilotus* species. Our study revealed that the genotypes and gene numbers were basically the same among the studied species, and no inverted repeats (IRs) were detected in the 12 chloroplast genomes. However, the presence of IRs was identified in another six species. This may be an uncommon occurrence in the conserved chloroplast genomes of the IRLC [35]. Recently, a reemergence of approximately 15 kb of novel IRs in *M. dentatus* and a parallel reappearance of approximately 9 kb of IRs in another species within the IRLC, *Medicago minima*, have been reported [36, 37]. Previous studies have shown that the IR region of chloroplasts is not as conserved as other regions and that the size of the IR could be highly variable [38]. Furthermore, some species have lost one copy of IR as same as *Melilotus*, including *Erodium texanum* (GenBank accession number HM125536) [38], *Cicer arietinum* (GenBank accession number NC_011163.1) [39], *Glycyrrhiza inflate* (GenBank accession number NC_042146.1) [40], *Medicago truncatula* (GenBank accession number NC_003119) [41], and three species from the genus *Astragalus* [42]. Our findings suggest that the differences in gene order in the chloroplast genomes among the 18 species are not unique but rather a common (Fig. 1). The inversion regions in these genomes were found to have a positive selection effect on the *rps18*, *rps7*, and *rpl16* genes, which may be the main cause of variation among the species. The *rps18* gene is crucial for chloroplast translation during plant development [43, 44]. The intergenomic inversion of chloroplasts and IR reemergence contributed to changes in the gene order, that is, chloroplast genome rearrangement and obvious chloroplast genome rearrangements have been observed in *Campanulaceae, Oleaceae* and *Geraniaceae* [38, 45, 46]. Genome rearrangement is often related to repetitive sequences, which can induce recombination, especially in some gymnosperms; because of IR deletion, gene recombination and deletion occur frequently [47]. The inverted fragments play a very important role in evolution; inverted fragments have resulted in the recombination of the chloroplast genome and contribute to the diversity of the chloroplast genome structure in *Pinaceae* [48, 49].

The analysis of entire chloroplast genomes allowed us to derive a highly reliable phylogenetic tree based on a set of 606 high-quality SNPs rooted with *M. truncatula* as an outgroup (Fig. 5c). Our phylogenomic framework is largely congruent with the phylogenetic framework of using ITS + *rbcL* + *matK*, *rbcL* + *matK* + *trnL-F* + ITS and the supermatrix of 70 EST-SSR markers [31–33]. These analyses all support the classification of species within the *Melilotus* genus into two groups. Apart from the analysis based on the concatenation of the four genes *rbcL* + *matK* + *trnL-F* + ITS, all other analyses endorse the clustering of *M. infestus*, *M. siculus*, *M. sulcatus*, and *M. speciosus*. In fact, the topology obtained based on rbcL + matK + trnL-F + ITS represents a multifurcating structure, indicating that the clustering of *M. speciosus* with the other three species cannot be rejected [32]. Notably, reports based on 47 L support these four species forming a monophyletic group. This finding is consistent with our results based on the complete chloroplast genome, although there may be differences in the specific interspecies relationships [50]. In contrast, in analyses based on EST-SSR markers and a small number of chloroplast genes, these four species clustered together with a few other species within the *Melilotus* genus [31]. In a phylogenetic tree based on LTRs, researchers also identified a monophyletic clade (clade II). This clade is composed of three species: *M. indicus*, *M. italicus*, and *M. spicatus* [50]. The clustering of these three species is also supported by analysis of EST-SSR markers, although the results indicate the inclusion of an additional species, *M. segetalis* [31]. The clustering of these four species, *M. indicus*, *M. italicus*, *M. spicatus*, and *M. segetalis*, is also supported to some extent by phylogenetic analysis based on a small number of chloroplast genes [32]. According to our study results, these four species are positioned at the base of this subgroup, forming paraphyletic groups along with other species within this subgroup. This finding highlights the distinctiveness of these four species. However, determining their precise phylogenetic position may necessitate the analysis of additional markers, such as whole-genome sequences. Comparing trees constructed from genomic SNPs and coding genes revealed good consistency, with only some differences among *M. atissimus, M. dentatus*, and *M. tauricus*, suggesting that it is possible to use both methods to identify the evolutionary relationships of species in *Melilotus* (Figs. 3 and 5c). The INVs we identified in this research could also be used as indicators to study the relationships among germplasms. In other words, species that have a close evolutionary relationship, such as *M. speciosus*, *M. infestus*, *M. siculus* and *M. sulcatus*. In these inversion regions, we also found that four specific genes (*rpoC2*, *clpP ycf2*, and *ycf1*) simultaneously accumulated high numbers of SNPs and InDels (≥ 20 bp) in the coding region (Fig. 5b). The *ycf2* gene around the INV is a putative ATPase with unknown function. This gene is expressed in many plants, including nonphotosynthetic plants. Previous experiments in tobacco indicated that this putative ATPase plays an essential role in cell survival via activity in the tobacco chloroplast [51]. These genes may serve as common hotspots of genetic variation in *Melilotus*, as indicated by this observation. Our research on the *Melilotus* pan-plastome shed light on the maternal inheritance in this genus and can be used as a basis for studying the phylogenetic degeneration of *Melilotus* with other species, constructing phylogenetic trees in the Fabaceae family, and even the Papilionoideae subfamily.

## Conclusions

This work is based on a comparative analysis of the chloroplast genomes of 18 *Melilotus* species and presents a comprehensive study of their evolutionary relationships and nucleotide substitution rates. The comparative genomic analysis was used to identify the genomic SNPs, InDels, main inversion positions, and evolutionary rate heterogeneity occurring in the chloroplast genomes of the studied *Melilotus* species. In total, 391 SNPs and 28 InDels located in exons and introns of 52 PCGs were found, and the results indicated that four specific genes (*rpoC2*, *clpP*, *ycf2*, and *ycf1*) simultaneously accumulated high numbers of SNPs and indels in the coding regions, while the other three specific genes (*rps18*, *rps7*, and *rpl16*) exhibited positive selection effects in the inversion regions; these distinctions may be the main source of variation among the 18 studied species.

## Materials and methods

### Plant materials and sequences

The raw sequence reads of the *Melilotus* accessions were download from National Center for Biotechnology Information [52]. To verify the accuracy of the chloroplast genome assembly, we also selected 16 *Melilotus* accessions for sequencing (Additional file 2: Table S1). These accessions were cultivated in a greenhouse on Yuzhong Campus, Lanzhou, China. Genomic DNA was extracted from fresh young leaves using the CTAB method. Sixteen paired-end libraries with an average insert size of 150 bp were constructed and sequenced using the BGISEQ-500 platform.

### Assembly and annotation of the chloroplast genome of *Melilotus*

GetOrganelle-1.7.1 [53] was employed to perform *de novo* assembly of the chloroplast genome, and the sequence was reordered to determine the assembly according to the alignment result on the basis of the comparison between the sequence and the *M*. *albus* reference chloroplast genome performed by MUMmer4.0 [54]. The gene features of the circular chloroplast DNA were annotated by GeSeq packages [55] and revised manually. Finally, all circular chloroplast genomes were constructed with OGRAW [56].

### Phylogenetic analysis

OrthoFinder was used to identify the gene families, and only the amino acid sequences of single-copy genes were used to construct the phylogenetic tree [57]. Multiple sequence alignments were performed with MUSCLE, and then a phylogenetic tree was constructed with both the maximum likelihood and Bayesian methods using IQ-TREE [58] and MrBayes [59] with 1000 times bootstrap replicates. *Medicago truncatula* (JX512023.1) was used as an outgroup to root the tree. All chloroplast genome sequences were aligned using Cactus [60] with the -auto option, and conserved blocks of the alignment were used to construct a phylogenetic tree with IQ-TREE. The tool iTOL was used to visualize and modify the tree file [61].

### Comparison and variant calling of the chloroplast genome

Chloroplast sequences of 17 other *Melilotus* species were aligned to the reference chloroplast genome of *M. albus* to perform genome comparisons using MUMmer4.0 [54], and structural variants were identified via smartie-sv [62]. The R package ggplot2 was used to visualize the mapping depths near the breakpoints.

### Nucleotide substitution rates

The nucleotide substitution rates, nonsynonymous rates (Dn), synonymous rates (Ds), and the ratio of nonsynonymous to synonymous rates (Dn/Ds), were determined using PAML v.4.9 [63]. Codon substitution models and likelihood ratio tests (codeml) were conducted based on the branch site model. The phylogeny generated was using the concatenated method. The “model = 0” option was used to allow single Dn/Ds values to vary among branches. The 76 PCGs were consolidated into nine groups (Table 2) to compare the different functions among the groups, such as photosystem I (PSA), photosystem II (PSB), the cytochrome B6f complex (PET), ATP synthase (ATP), rubisco large subunit (Rubisco), RNA polymerase (RPO), ribosomal proteins large subunit (RPL), ribosomal proteins small subunit (RPS) and NADH dehydrogenase (NDH), and other genes (OG, including *ycf1*, *ycf2*, *ycf3*, *ycf4*, *accD*, *clpP*, *ccsA*, *cemA* and *matK*).

### Variant calling and construction of the phylogenetic tree

The data for the *Medicago archiducis* sample were downloaded from NBCI (SRX9404272), and bwa [64] was used to align the clean reads, which included 18 *Melilotus* and *Medicago archiducis* reads, to the *M. albus* chloroplast genome. The alignment files were sorted and indexed with SAMtools [65]. Variant calling was performed using GATK-4.0 [66] (https://gatk.broadinstitute.org) with default parameters, and variants were filtered by VCFtools (http://vcftools.sourceforge.net) with the parameters “--min-alleles 2 --max-alleles 2 --min-meanDP 5 --maf 0.05 --max-missing 0.5”. The filtered mutations were annotated using ANNOVAR [67]. The filtered SNPs were used to analyse the phylogenetic tree, and IQ-TREE [58] was used to select the best model and construct a phylogenetic tree using the maximum likelihood method with 1000 times bootstrap. The online iTOL [61] was used to visualize the phylogenetic tree.

### Electronic supplementary material

Below is the link to the electronic supplementary material.

Supplementary Material 1

Supplementary Material 2

Supplementary Material 3

Supplementary Material 4

## Data Availability

The raw sequence reads of the *Melilotus* accessions were obtained from National Center for Biotechnology Information under the BioProject accession numbers PRJNA781345 and PRJNA759778. *Melilotus* accessions were applied from the National Plant Germplasm System (NPGS, United States Department of Agriculture, USA). The application procedure is legal. Plant germplasm is distributed to scientists, educators, producers and other bona fide research and education entities from U.S. National Plant Germplasm System (NPGS) active collection sites.
