# Supplementary material for: A comparative plastome approach enhances the assessment of genetic variation in the Melilotus genus
Source: BMC Genomics. 2024 Jun 3;25:556. doi: 10.1186/s12864-024-10476-y (PMC11149310; doi:10.1186/s12864-024-10476-y)
Supplement: Supplementary file 1 — Supplementary Material 1 [file 12864_2024_10476_MOESM1_ESM.docx]

Additional file1: This file contains all of the additional material (Figure S1, S2 and S3) associated with the manuscript.


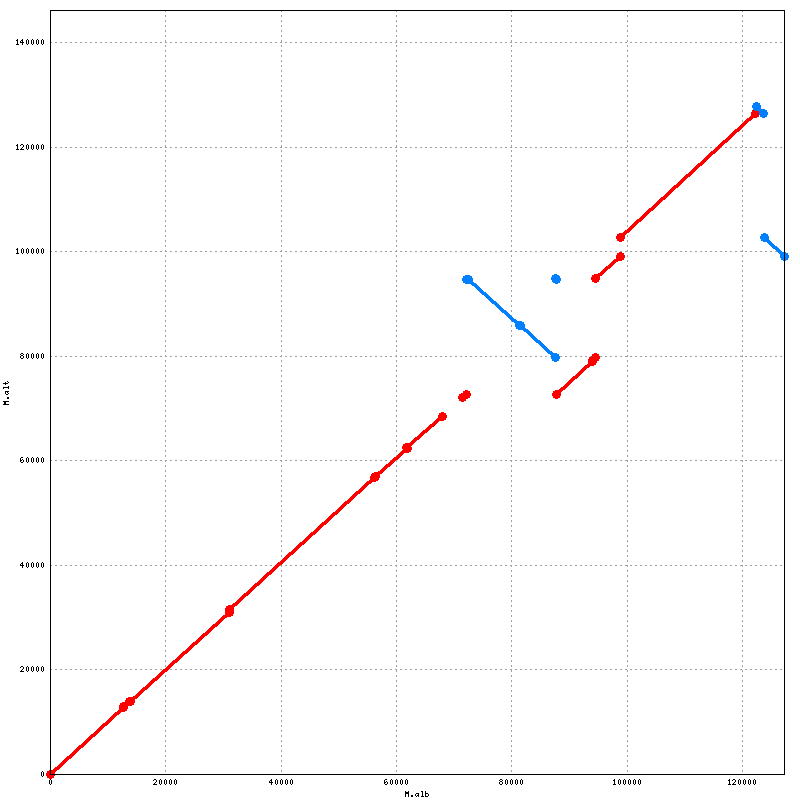

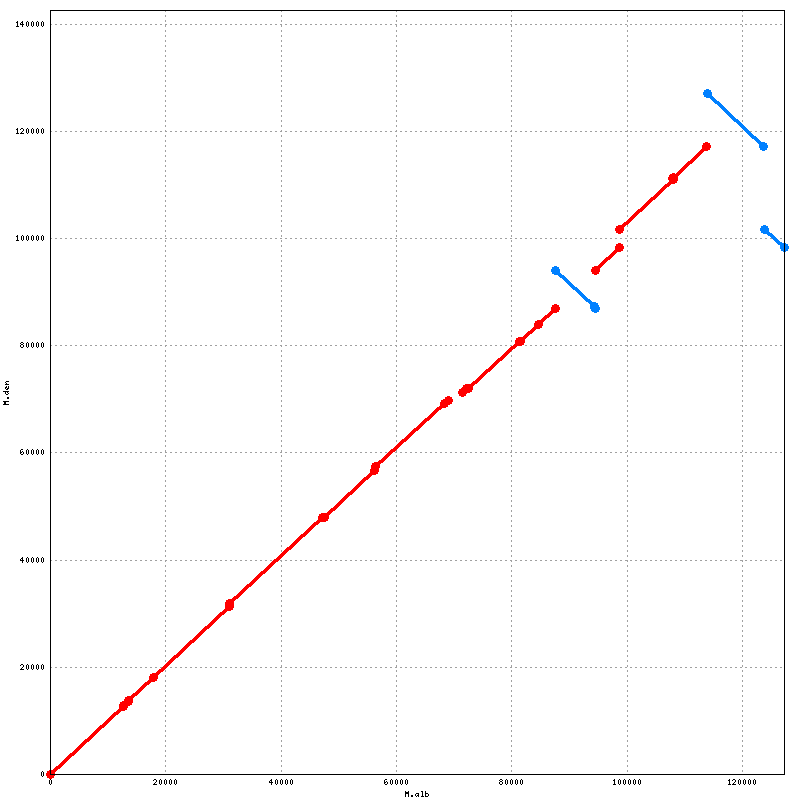

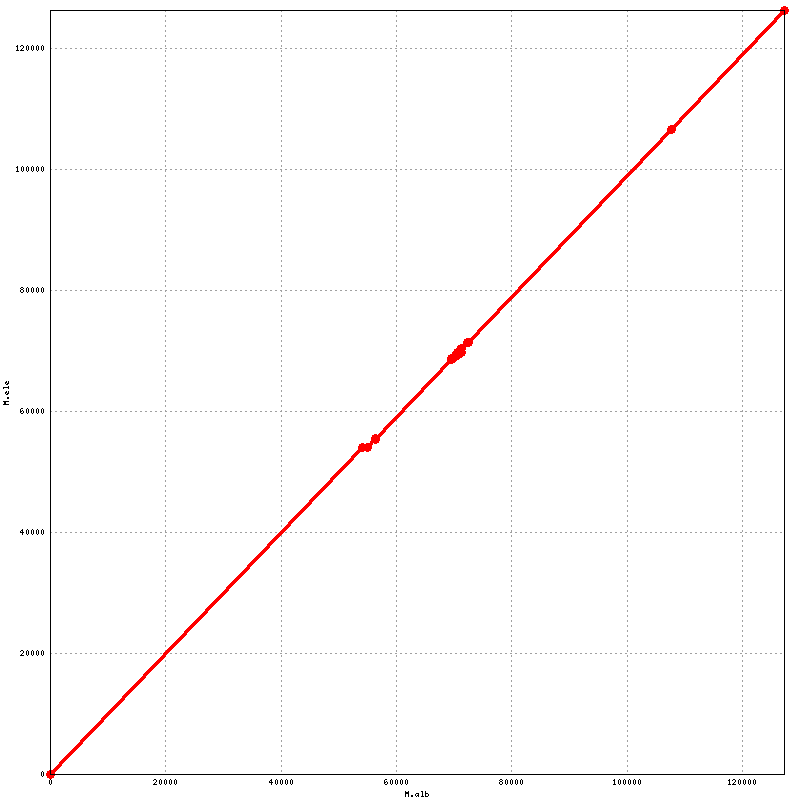


M.alt_vsM.alb M.den_vs_M.alb M.ele_vs_M.alb


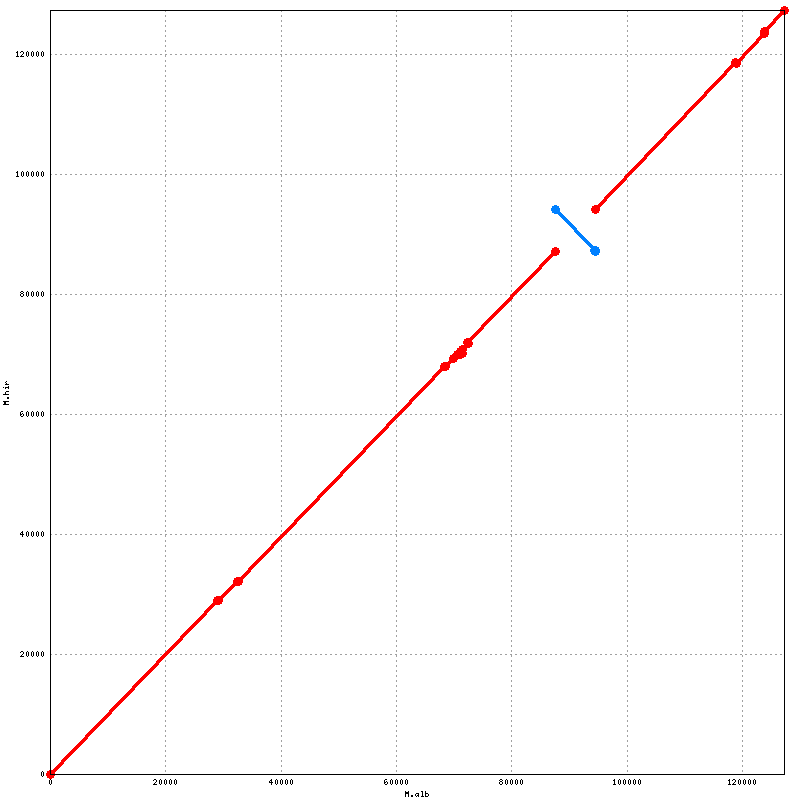

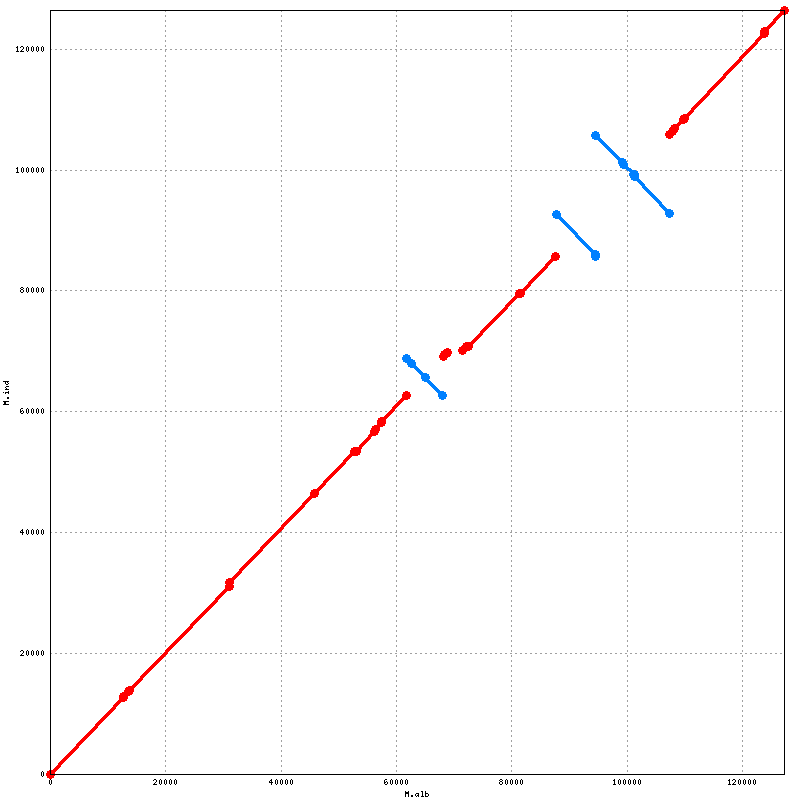

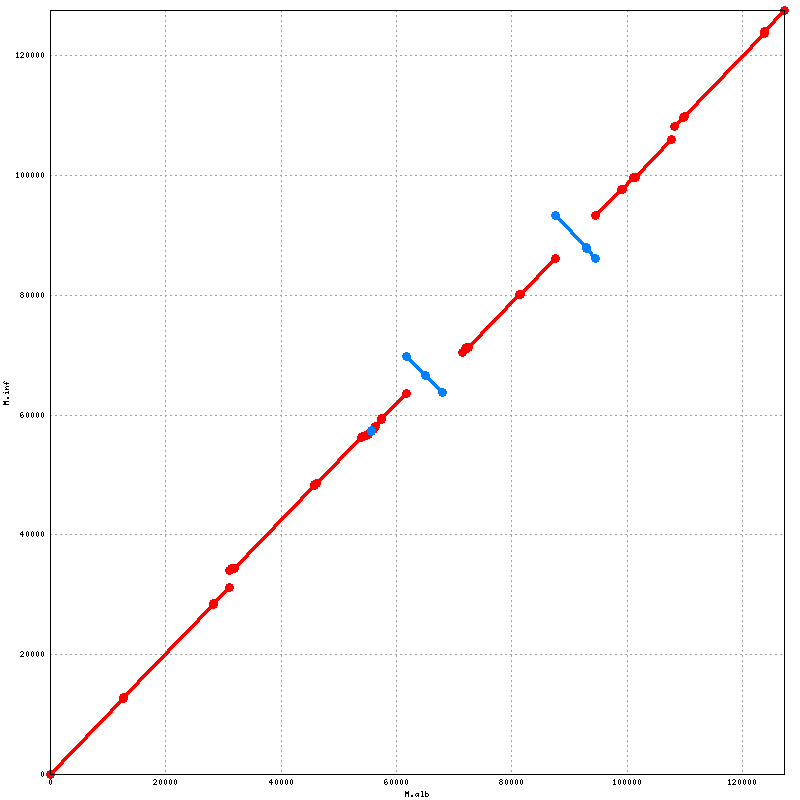


M.hir_vs_M.alb M.ind_vs_M.alb M.inf_vs_M.alb


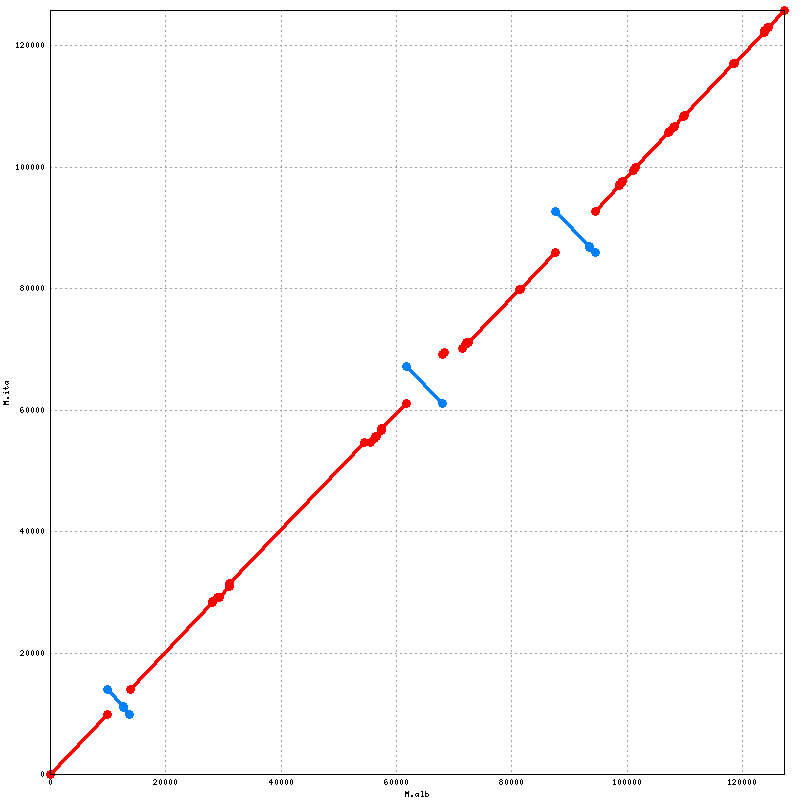

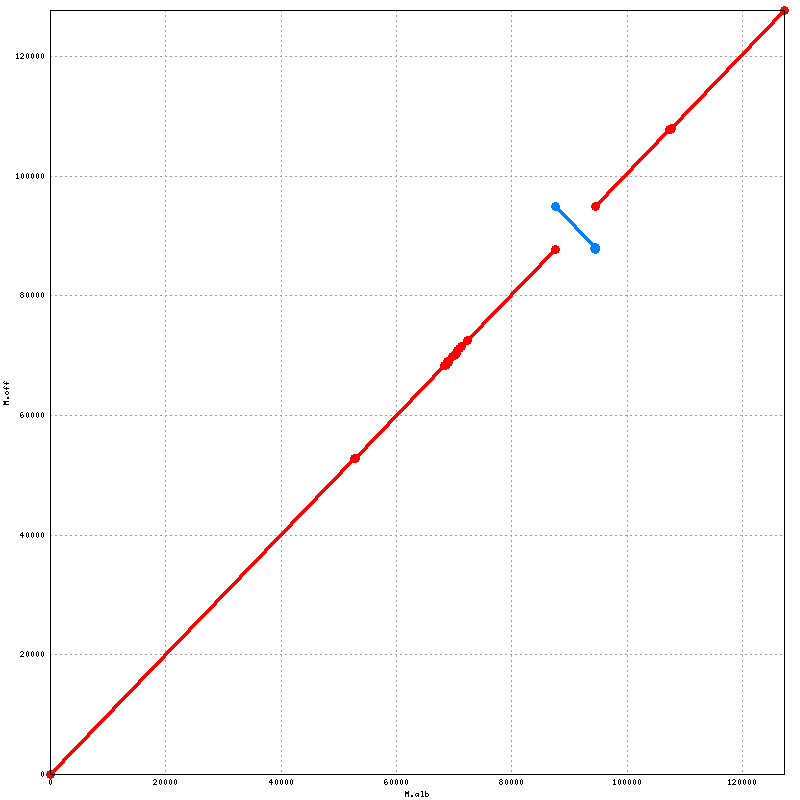

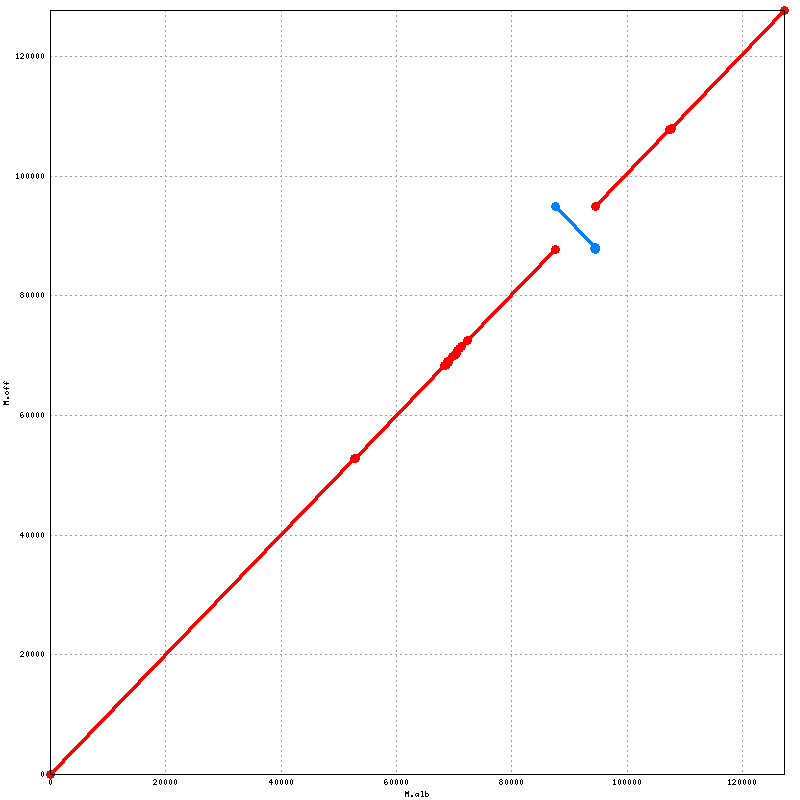


M.ita_vs_M.alb M.off_vs_M.alb M.pol_vs_M.alb


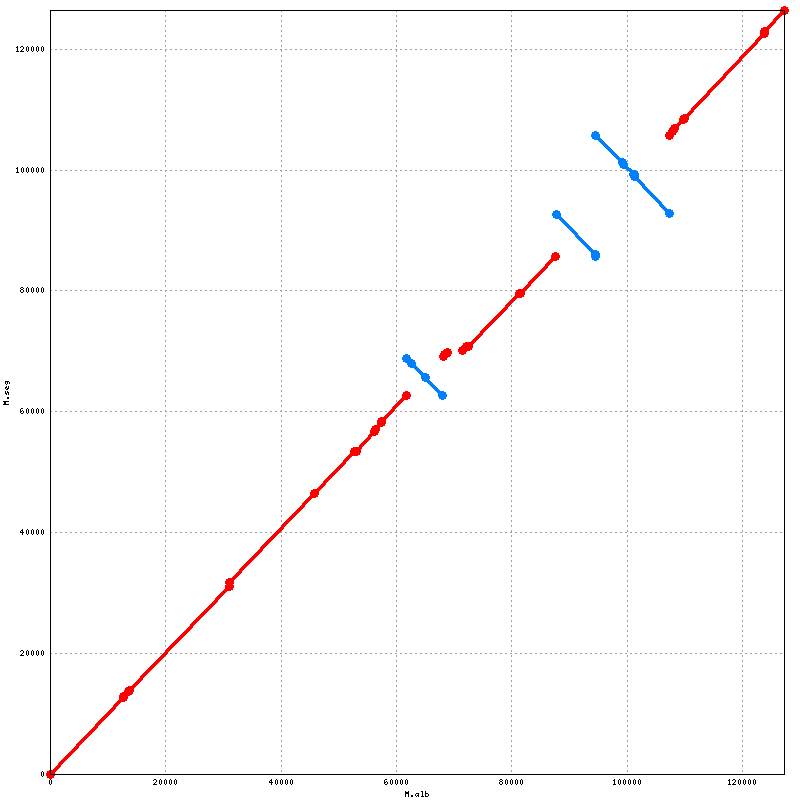

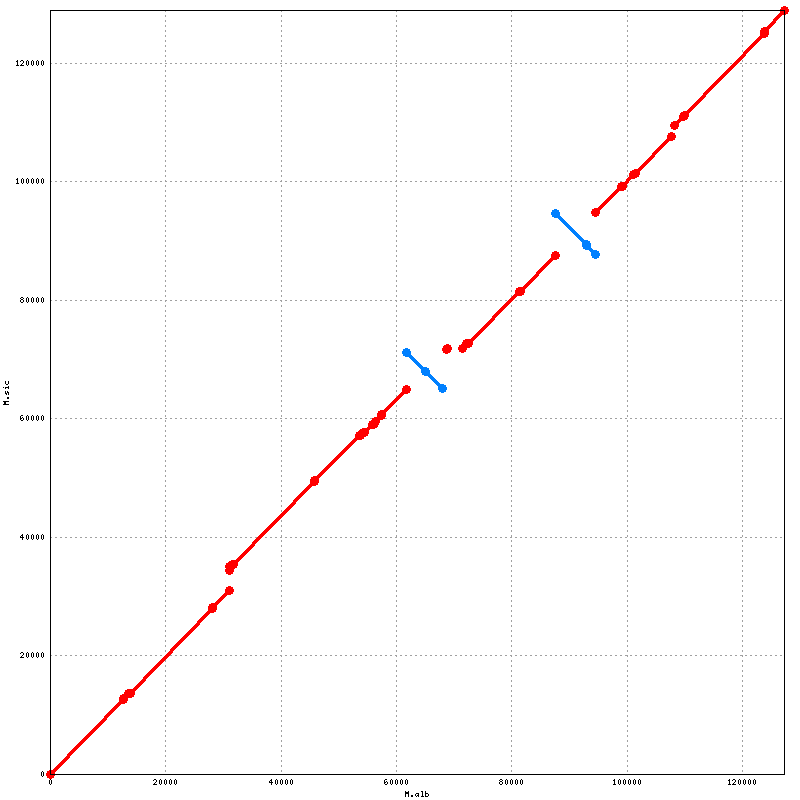

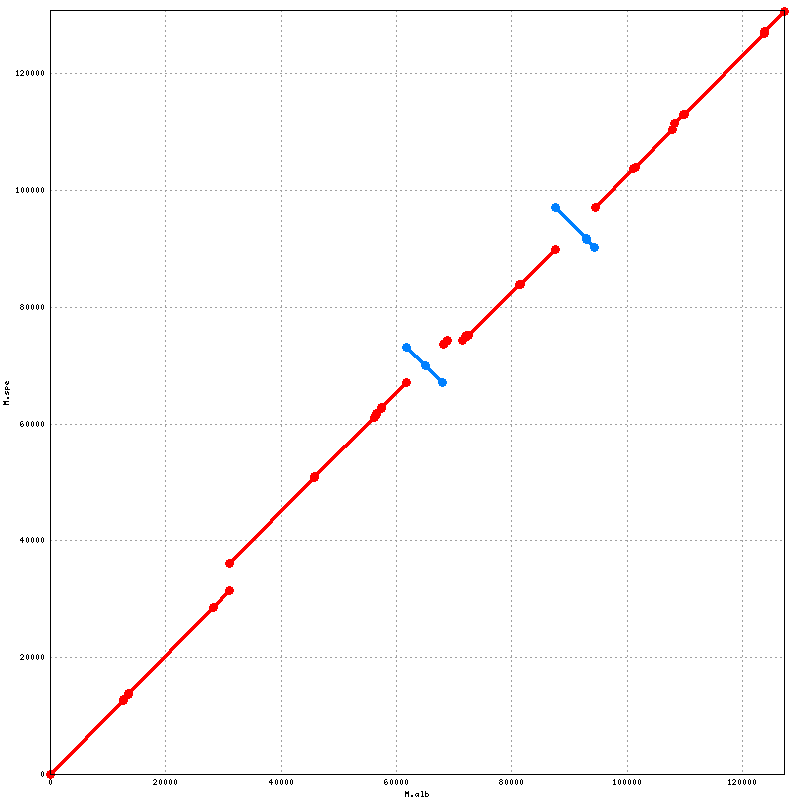


M.seg_vs_M.alb M.sic_vs_M.alb M.spe_vs_M.alb


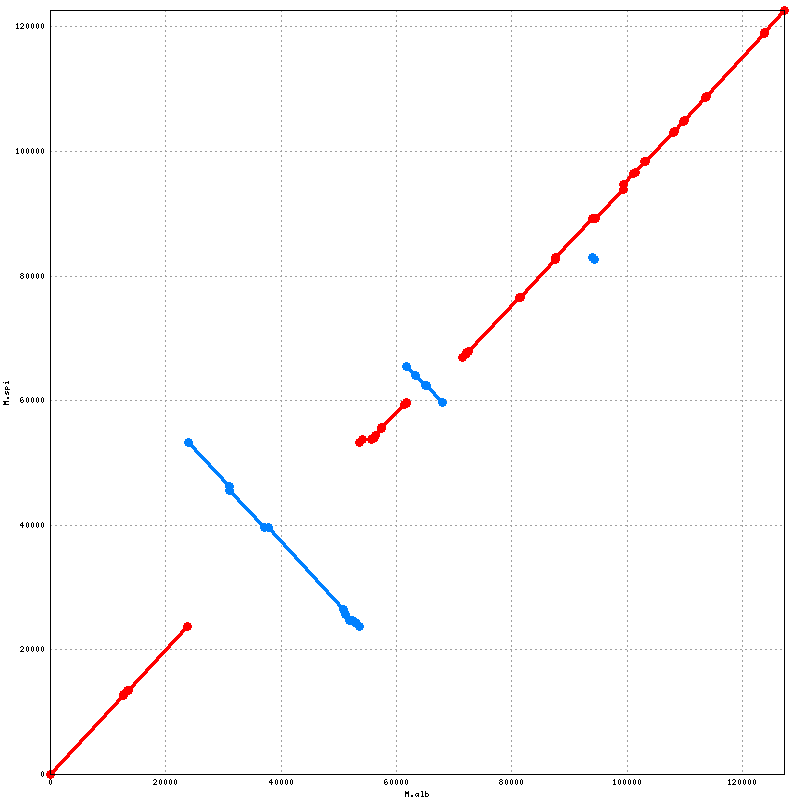

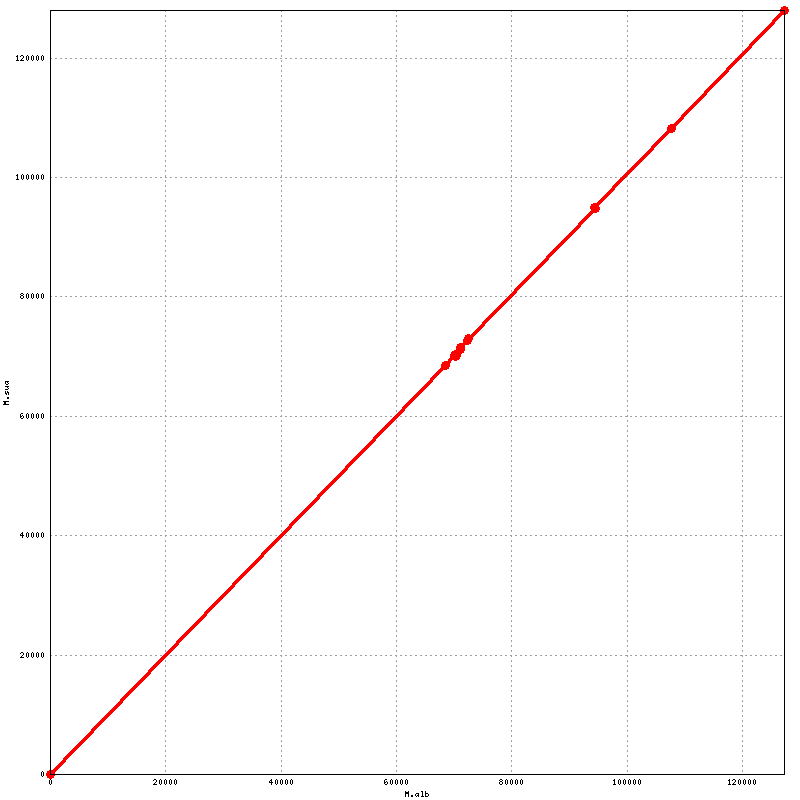

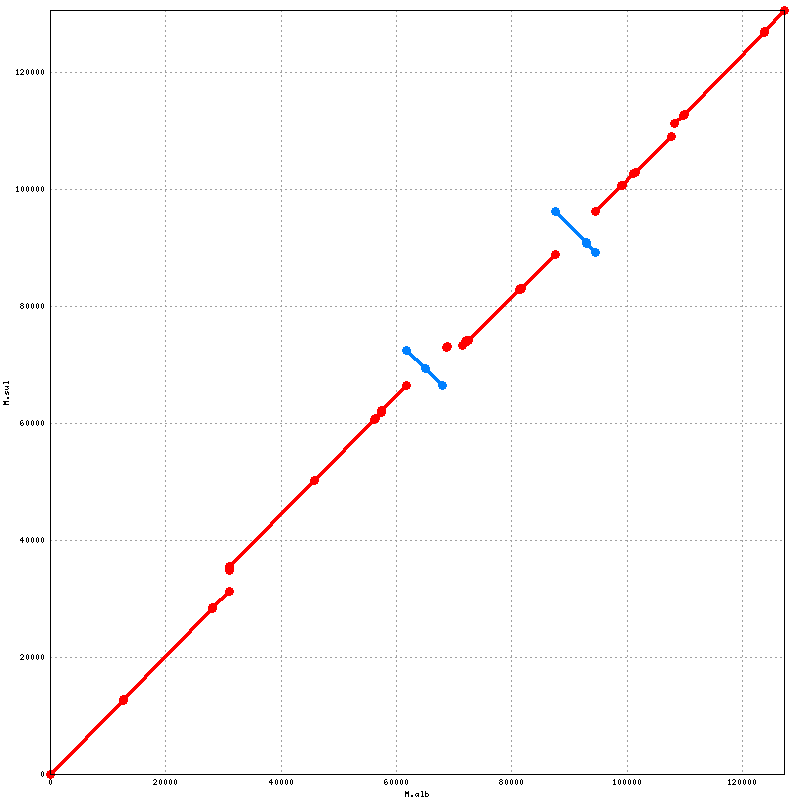


M.spi_vs_M.alb M.sua_vs_M.alb M.sul_vs_M.alb


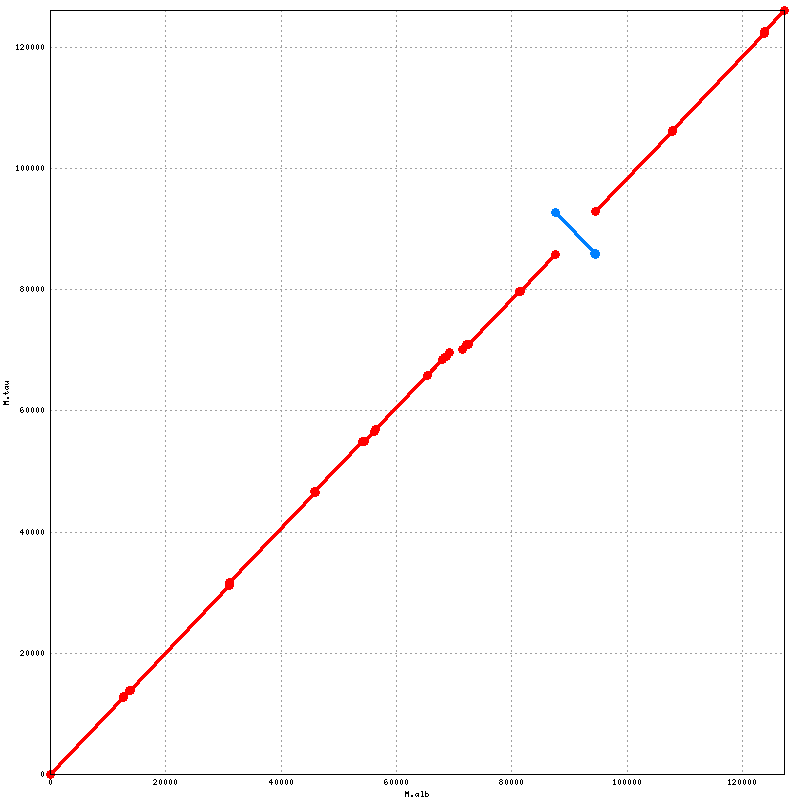

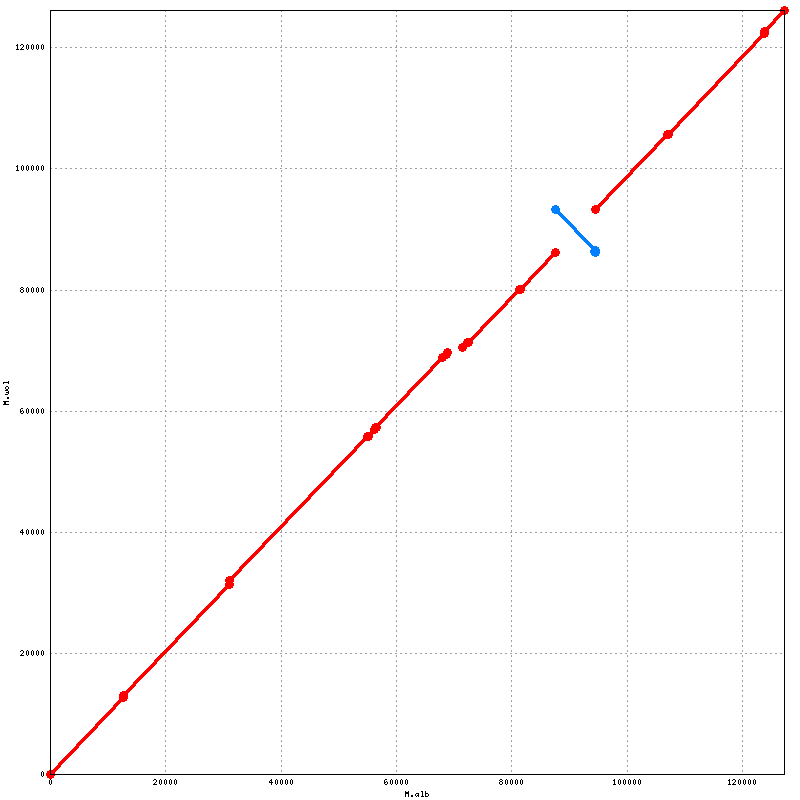


M.tau_vs_M.alb M.wol_vs_M.alb

**Figure S1.** Number collinear plot between *M. albus* and other species


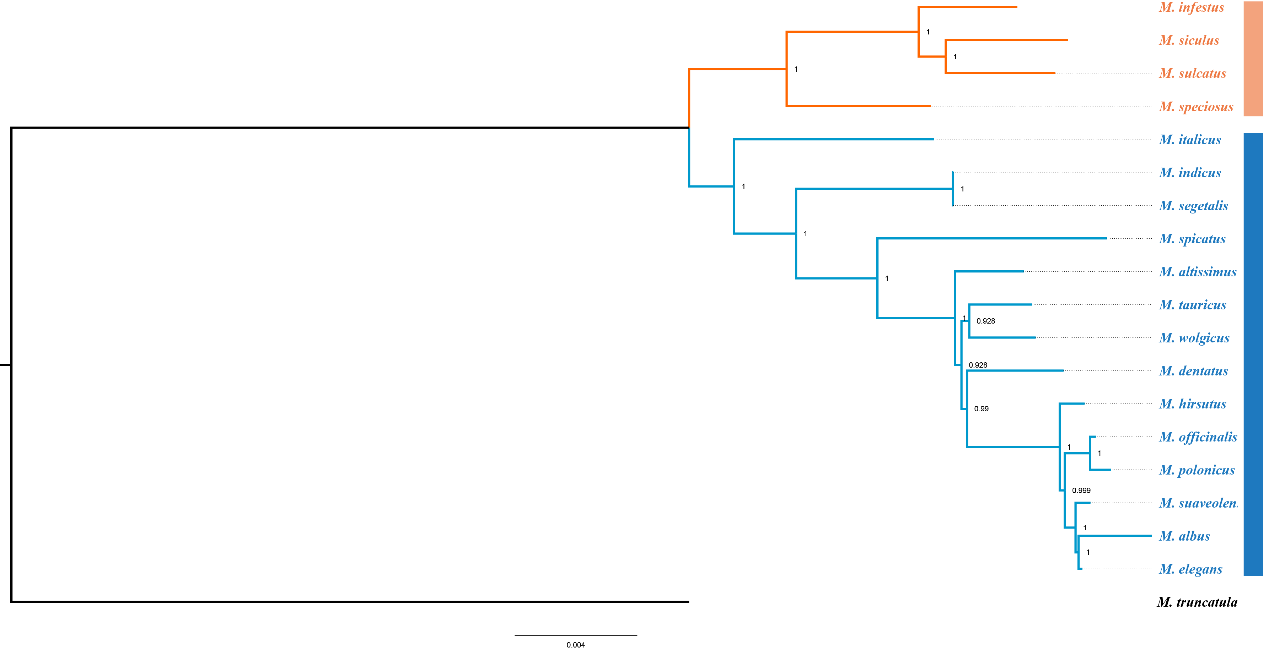


**Figure S2.** Phylogenetic tree constructed by single copy genes of 18 species use MrBayes


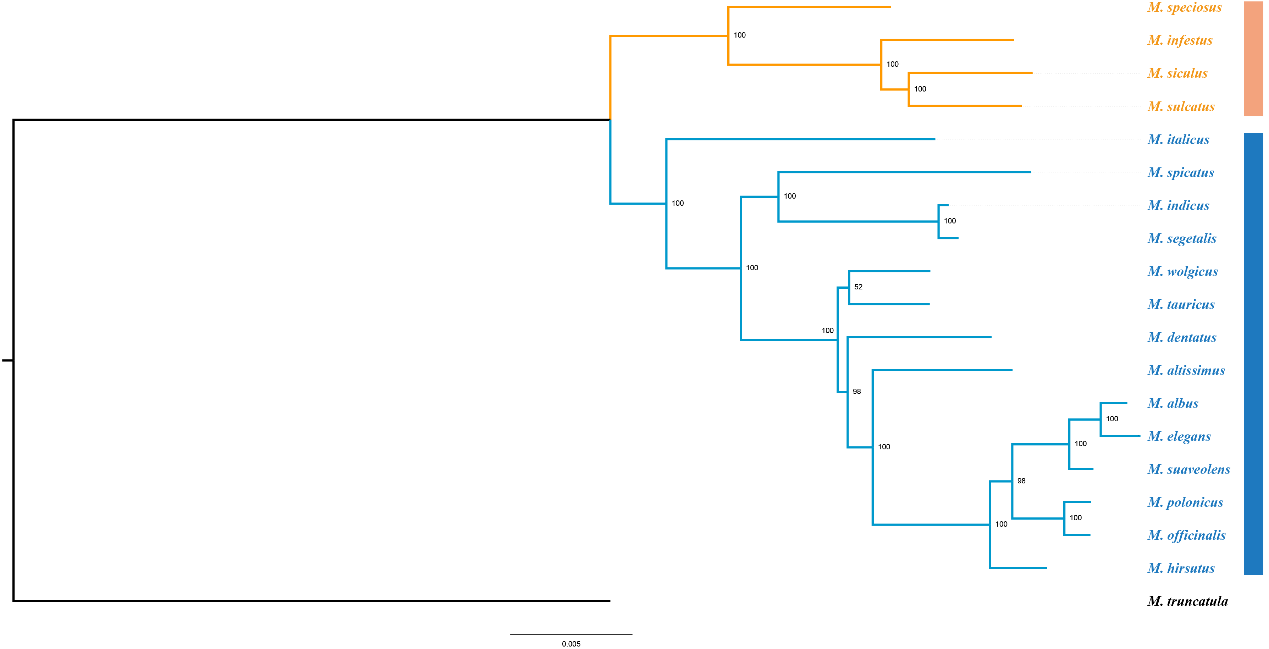


**Figure S3.** Phylogenetic tree constructed by chloroplast genome sequence of 18 species use IQTREE
